# Mitochondrial trifunctional protein deficiency caused by a deep intronic deletion leading to aberrant splicing

**DOI:** 10.1002/jmd2.12459

**Published:** 2024-12-16

**Authors:** Thomas Cassini, Sarah Silverstein, Molly Behan, Cynthia J. Tifft, May Christine Malicdan, David R. Adams, Undiagnosed Diseases Network, Sun‐Young Ahn, Debra S. Regier

**Affiliations:** ^1^ Division of Medical Genetics and Genomic Medicine, Department of Pediatrics Vanderbilt University Medical Center Nashville Tennessee USA; ^2^ Neuromuscular and Neurogenetics Diseases of Childhood Section NINDS, NIH Bethesda Maryland USA; ^3^ NIH Undiagnosed Diseases Program, NIH Intramural Research Program NIH Bethesda Maryland USA; ^4^ Rutgers New Jersey Medical School Rutgers University Newark New Jersey USA; ^5^ Medical Genetics Branch, National Human Genome Research Institute National Institutes of Health Bethesda Maryland USA; ^6^ Division of Pediatric Nephrology Children's National Hospital Washington District of Columbia USA; ^7^ The George Washington School of Medicine and Health Sciences George Washington University Washington District of Columbia USA; ^8^ Division of Genetics and Metabolism Children's National Hospital Washington District of Columbia USA

**Keywords:** intronic variant, nephrotic syndrome, RNAseq, trifunctional protein deficiency, Undiagnosed Diseases Network

## Abstract

Trifunctional protein deficiency (TFP) is a disorder of fatty acid beta‐oxidation associated with metabolic, cardiac, and liver dysfunction in severe forms. We present two siblings diagnosed by newborn screening and confirmed by biochemical testing at birth. Their clinical course was complicated by recurrent rhabdomyolysis, retinopathy, and hypoparathyroidism. Both siblings were also diagnosed with focal segmental glomerulosclerosis (FSGS) and bone marrow failure and ultimately died of hypoxemic respiratory failure. Initial sequencing of the TFP‐associated genes *HADHA* and *HADHB* showed only a paternally inherited variant in *HADHB,* NM_000183.3:c.1059del (p.Gly354AspfsTer10). Subsequent evaluation by the Undiagnosed Diseases Network with genome and transcriptome sequencing revealed a rare maternally inherited 17 base pair deletion in *HADHB*, NM_000183.3:c.1390‐515_1390‐499del, located in the final intron and resulting in a pseudoexon that harbors a premature termination codon. Both sisters were compound heterozygous for this and the paternal premature termination codon. No other variants were detected that were potentially causative for the FSGS and bone marrow failure on genome sequencing. A review of the literature at that time revealed several case reports of the uncommon clinical findings of FSGS, bone marrow failure, and pulmonary involvement in patients with TFP, confirming this clinical diagnosis as the complete explanation for these siblings.

**Synopsis**

RNAseq helped to identify a deep intronic variant in trans with a pathogenic variant to make a molecular diagnosis of trifunctional protein deficiency in two siblings with a complicate clinical presentation.

## 1 INTRODUCTION

Trifunctional protein deficiency (TFP, MIM# 609015) is a disorder of impaired mitochondrial beta‐oxidation of long‐chain fatty acids.^1^ TFP is caused by biallelic loss of function variants in one of the two genes, *HADHA* or *HADHB*.^2^, ^3^ The three enzymatic activities of the mitochondrial trifunctional protein complex are long‐chain enoyl‐CoA hydratase, long‐chain 3‐hydroxyacyl‐CoA dehydrogenase, and long‐chain 3‐ketoacyl‐CoA thiolase.^4^ This is closely related to long‐chain acyl‐CoA dehydrogenase (LCHAD) deficiency, which is due to an isolated deficiency of the second TFP activity.^5^

The typical presentation of TFP shares some features with other fatty acid oxidation disorders, including hypoketotic hypoglycemia, rhabdomyolysis, and cardiomyopathy. Chronic progressive complications unique to TFP include polyneuropathy, hepatomegaly, and retinopathy.^6^ TFP was traditionally diagnosed by acylcarnitine profile testing, with higher sensitivity during an acute decompensation. A positive diagnosis is supported by elevated long‐chain and long‐chain hydroxyl carnitine ester elevations.^7^ Molecular genetic testing is increasingly used to confirm the diagnosis. TFP has been included as a target for expanded newborn screening, with the goal of reaching a diagnosis prior to the onset of symptoms.^8^, ^9^ Early diagnosis enables therapies such as dietary modification and medium‐chain triglyceride supplementation to be instituted proactively, potentially ameliorating or preventing acute decompensation episodes and improving disease management.^10^

**FIGURE 1:**
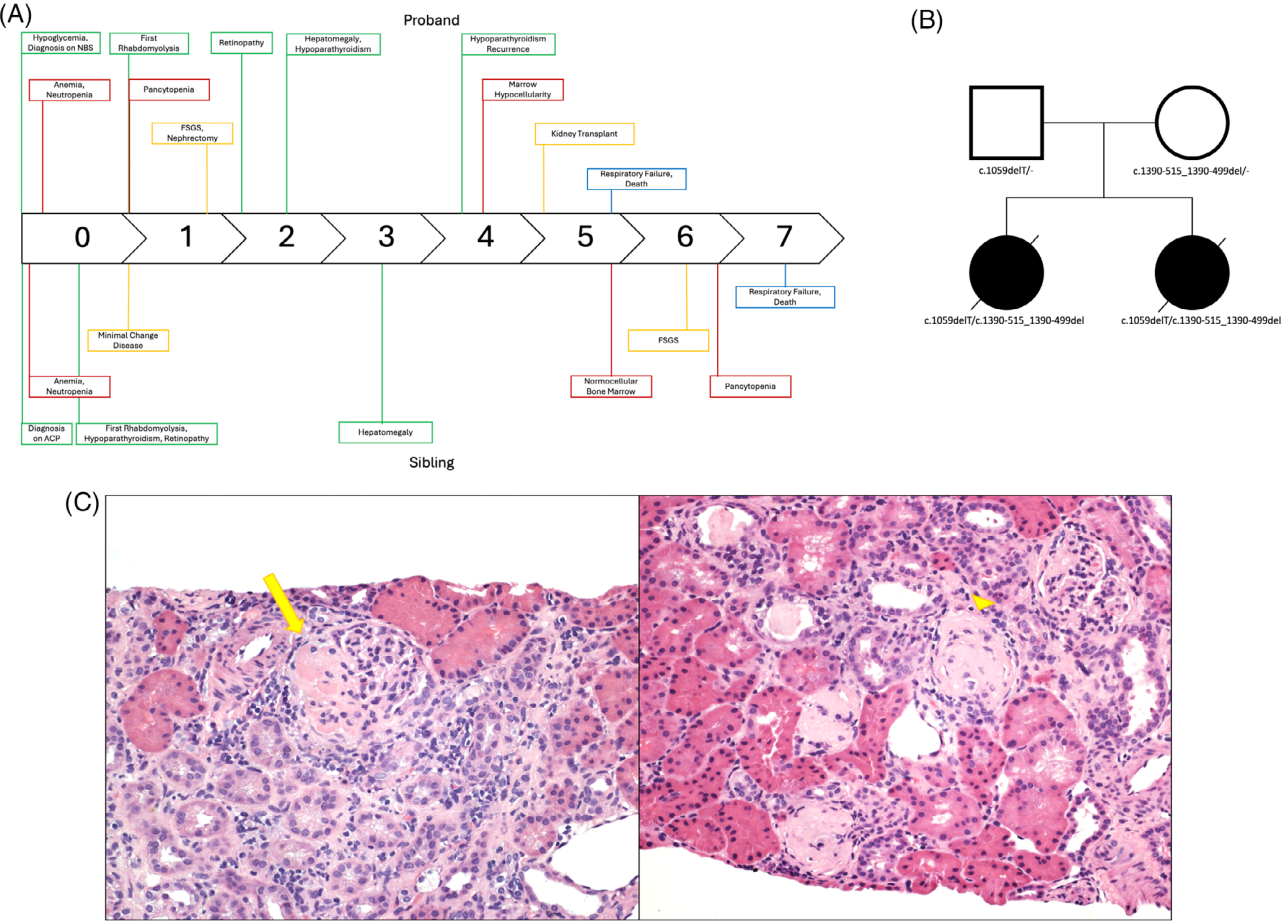
Clinicopathologic features of TFP. (A) Timeline of the clinical features for the proband and her sibling. Each had some typical features of TFP along with hematologic, renal, and respiratory involvement. (B) Pedigree illustrating that both siblings are affected due to compound heterozygous variants in *HADHB* (NM_000183.3). (C) Light micrograph of hematoxylin and eosin stained renal biopsy sections showing FSGS (left, arrow) and global glomerulosclerosis (right, arrow head). ACP, acylcarnitine profile; FSGS, focal segmental glomerulosclerosis; NBS, newborn screen.

We report two siblings with biochemical confirmation of TFP. Their molecular diagnosis was complicated by the detection of only one likely disease‐causing variant in *HADHB*. While some clinical features present in these siblings were typical of TFP, they also developed some features that raised concern for a second diagnosis (Figure 1A). Due to these unusual clinical features and the lack of a confirmed genetic diagnosis, the siblings were therefore enrolled in the National Institutes of Health (NIH) Undiagnosed Diseases Program (UDP) for further evaluation. RNA sequencing and review of the updated literature helped to identify the second variant, confirm the diagnosis, and further support previous reports of an expanded phenotype for TFP (Figure 1B).

## 2 METHODS

### 2.1 Undiagnosed Diseases Program enrollment

The proband was admitted to the NIH Clinical Center and enrolled in protocol 15‐HG‐0130, “Clinical and Genetic Evaluation of Patients with Undiagnosed Disorders Through the Undiagnosed Diseases Network,” approved by the NHGRI Institutional Review Board.^11^ The participant's parents gave written, informed consent. Peripheral whole blood samples were collected, and DNA was extracted using standard methodologies. Genome sequencing was performed on the proband, her parents, and her sister at the Baylor Genetics Sequencing Center (Houston, TX) through the Undiagnosed Diseases Network.^12^, ^13^, ^14^

### 2.2 RNA‐seq analysis

RNA sequencing was performed on RNA extracted from dermal fibroblasts derived from the proband and her affected sibling. Briefly, cells were grown to confluency and adherent cells were collected from T75 flasks through trypsinization using TrypLE (A1285901, Gibco/Thermofisher), washed with PBS and pelleted. Total RNA was extracted using Maxwell® RSC (AS4500, Promega) and Maxwell® RSC simplyRNA Cells Kit (AS1390, Promega) following manufacturer's instructions. The concentrations were determined by Qubit (Thermofisher) and the quality of RNA was determined by Bioanalyzer. RNAseq was done at the Baylor Genetics Sequencing Center. Reads were aligned to GRCH38 using the GTEx V10 pipeline (https://github.com/broadinstitute/gtex-pipeline) and inspected manually using the Integrative Genomics Viewer (IGV) to identify causal variants.^15^ The proband BAM file was included in a cohort comprising ~100 undiagnosed rare disease patients and analyzed with OUTRIDER to identify decreased transcript expression.^16^ As OUTRIDER runs best when samples contain similar read depth, the sibling BAM file was unable to be included as QC of the bam files with RNAseqC demonstrated a much higher read depth (~400 M) compared with the rest of the cohort (~200 M reads/sample) and a disproportionate strand specificity compared with other cohort samples (Figure S2). Sashimi plots were generated using ggSashimi.^17^ Control samples included healthy samples from GTEx and disease controls from our cohort.

### 2.3 Reverse transcriptase polymerase chain reaction (RT‐PCR)

In addition to the dermal fibroblasts from the proband and her affected sibling, control fibroblasts were purchased from ATCC and Coriell. Cells were grown to confluency and RNA was extracted as above. The RNA was purified of residual DNA using the DNA‐*free* DNA Removal Kit (AM1906, Thermo Fisher), and the concentration was determined by nanodrop. RNA to cDNA reverse transcription was performed using High‐Capacity RNA‐to‐cDNA Kit (4387406, Thermo Fisher) using 0.4 μg RNA per reaction. The cDNA was amplified by PCR using AmpliTaq Gold (JP3772, Roche) and a forward primer flanking exon 10: 5′‐AGTGACAGCTGCAAATTCTTCT‐3′ with a reverse primer flanking exon 16: 5′‐CAACCACCCACACATCTCATGTG‐3′. The PCR followed a standard touchdown protocol spanning Tms of 60.5–70°C.

### 2.4 Gel electrophoresis and cloning

Using a Bio‐Rad PowerPac (1645050, Bio‐Rad), a 1% agarose gel was run at 100 V for 45 min to separate RT‐PCR amplicons. The gel‐bands were visualized using Syngene U:Genius3 gel documentation system.

RT‐PCR product was cloned into the TOPO TA cloning vector (K4575J10, Thermo Fisher) and transformed unitlizing OneShot™ TOP10 Chemically Competent *Escherichia coli* (C404003, Thermo Fisher). Colonies were picked from both the proband and affected sibling (~50 colonies each) and the cDNA was amplified. All the amplified clones were screened on a 1% agarose gel and cDNA associated with bands of varying size were sent for Sanger sequencing (Psomagen, Inc., Rockville, MD) with the M13 primer.

## 3 RESULTS

### 3.1 Proband case description

The proband was a girl born at 38 and 3/7 weeks of gestation following a pregnancy complicated by maternal liver enzyme elevation. On Day 2 of life, she developed hypoglycemia with a glucose of <10 mg/dL and lactic acidosis. Her newborn screen collected on Day 2 of life showed the following elevations: C14 1.78 nmol/mL (normal <0.70), C14 1 1.06 nmol/mL (<0.66), C16‐OH 2.99 nmol/mL (normal <0.10), and C18‐OH 0.99 nmol/mL (<0.11). This pattern was consistent with a diagnosis of TFP. Free and total carnitine, liver enzymes, creatine kinase (CK), and echocardiogram were within normal limits. While feeding was initiated with a combination of low‐fat formula and breast milk, she was supported with dextrose‐containing fluids. However, due to poor feeding, a gastrostomy tube was placed at 3 weeks of life. With this feeding regimen, she was metabolically stable without hospital admissions during the first year of life. Her growth and development were typical, excluding mild delays in the gross motor domain.

During the second year of life, her clinical course became more complicated with multiple acute decompensations, and several unusual complications are presented. At the age of 1 year, she was admitted to the pediatric ICU for a febrile illness with vomiting. Her CK was elevated at 4377 U/L and liver enzymes were elevated with an AST of 194 U/L and an ALT of 324 U/L. Additionally, she was found to be anemic with a hemoglobin (Hgb) of 9.0 g/dL and neutropenic with a total white blood cell count of 5.7 × 10^3^/μL and absolute neutrophil count (ANC) of 400/μL. Acylcarnitine profile around 1 year of age showed the following elevations: C14‐OH 0.05 (normal <0.04), C16‐OH 0.34 (normal <0.06), C18 0.18 (normal 0.01–0.11), C18:2 0.41 (normal <0.3), C18:1‐OH 0.25 (normal <0.03), and C18:2‐OH 0.34 (normal <0.05). Review of the history showed that her Hgb and ANC were normal at birth but were below the lower limit of normal by 2.5 months of age. Evaluation for additional causes of the cytopenia was non‐revealing. She did have a response to granulocyte colony stimulating factor administration with improved ANC. She had multiple similar hospitalizations during the second year of life.

At 19 months of age, she developed anasarca and was diagnosed with nephrotic syndrome. Laboratory evaluation showed an albumin of 0.6 g/dL and a urine protein‐to‐creatinine ratio of 18.3. Subsequent renal biopsy revealed pathology consistent with focal segmental glomerulosclerosis (FSGS) (Figure 1C). She was treated with intravenous albumin, diuretics, and fluid restriction. Her serum creatinine worsened and she was initiated on hemodialysis and transitioned to peritoneal dialysis. She underwent bilateral nephrectomies due to persistent proteinuria and eventually received a living, unrelated donor kidney transplant at the age of 5.

The proband also exhibited pancytopenia and eventually became transfusion‐dependent for treatment of anemia. She showed no response to erythropoietin nor improvement following renal transplant. Her leukopenia fluctuated and was predominantly driven by neutropenia. Her T‐cell and B‐cell subsets were normal, and she had intermittent hypogammaglobulinemia. She underwent multiple bone marrow biopsies showing progressive hypocellularity, from 80%–90% at age 2 to 40%–50% at age 5.

She developed additional complications known to be associated with TFP. At 28 months of age, she was found to have retinopathy with diffuse retinal pigmentary changes with macular sparing and early choroidoretinal atrophy. Hepatomegaly with coarse echotexture was noted on ultrasound at 2 years of age with a liver span of 11.5 cm. A repeat ultrasound at 5 years of age was stable with a liver span of 12 cm. She had fluctuating primary hypoparathyroidism, which was first noted at 2 years of age, improved and then recurred at 4 years of age. She did not have significant cardiomyopathy, however, concentric left ventricular hypertrophy with mild left ventricular dilation at 2 years of age, which improved without treatment.

At age 5, she presented to the emergency department with fever, cough, and hypoxemia. Her computed tomography scan showed ground‐glass opacities and a bronchoalveolar lavage showed lipid and hemosiderin laden macrophages. Infectious evaluations were negative. She was hospitalized 1 month later with recurrent hypoxemia. She had an increasing Epstein–Barr virus titer and was treated with Rituximab. One month after this, she was again hospitalized and required increasing respiratory support with bilevel‐positive airway pressure and escalated to endotracheal intubation. She eventually died of hypoxemic respiratory failure. Autopsy showed extensive, severe, acute to chronic diffuse alveolar damage and interstitial pneumonia. All infectious investigations were negative.

### 3.2 Sibling case description

The proband's sister, who was 1 year younger than the proband, was also diagnosed with TFP on an acylcarnitine profile obtained on cord blood.^18^ This showed the following elevations: C14‐OH 0.14 nmol/mL (normal <0.04), C16‐OH 0.50 nmol/mL (normal <0.10), C18:1‐OH 0.39 nmol/mL (normal <0.03), and C18:2‐OH nmol/mL 0.23 (normal <0.04). She had recurrent rhabdomyolysis typically triggered by viral illnesses. Around 1 month of age, she was diagnosed with anemia and neutropenia that progressed to involve other cell lines with eventual pancytopenia. At 9 months of age, she developed nephrotic‐range proteinuria, and a renal biopsy showed findings consistent with FSGS, that was initially responsive to tacrolimus. Acylcarnitine profile around 1 year of age showed the following elevations: C14 0.21 nmol/mL (normal 0.01–0.14), C14:2 0.15 nmol/mL (normal 0.01–0.12), C14‐OH 0.10 nmol/mL (<0.04), C16 0.83 nmol/mL (normal 0.04–0.51), C16:1 0.26 nmol/mL (normal <0.2), C16‐OH 0.72 nmol/mL (normal <0.06), C18 0.43 nmol/mL (0.01–0.11), C18:1 0.58 nmol/mL (normal 0.03–0.44), C18:2 0.58 nmol/mL (normal <0.3), C18:1‐OH 0.53 nmol/mL (normal <0.03), and C18:2‐OH 0.41 nmol/mL (normal <0.05). She also developed retinopathy. At age 7, she passed away from hypoxemic respiratory failure similar to her sister.

### 3.3 DNA sequencing identifies one variant in HADHB

The proband underwent sequencing of *HADHA* and *HADHB* after the newborn screening result suggested TFP biochemically. No pathogenic variants, likely pathogenic variants, or variants of uncertain significance in *HADHA* were observed. One paternally inherited variant was found in *HADHB*: NM_000183.3:c.1059delT (p.G354Dfs*10), which is located in exon 13 of 16 and is predicted to undergo nonsense mediated decay. This variant is absent in gnomAD v4.0.0.^19^ Based on American College of Medical Genetics guidelines, this variant is classified as pathogenic based on meeting the PVS1, PM2_supporting, and PP4 criteria.^20^, ^21^

### 3.4 RNAseq reveals a second variant in HADHB

**FIGURE 2:**
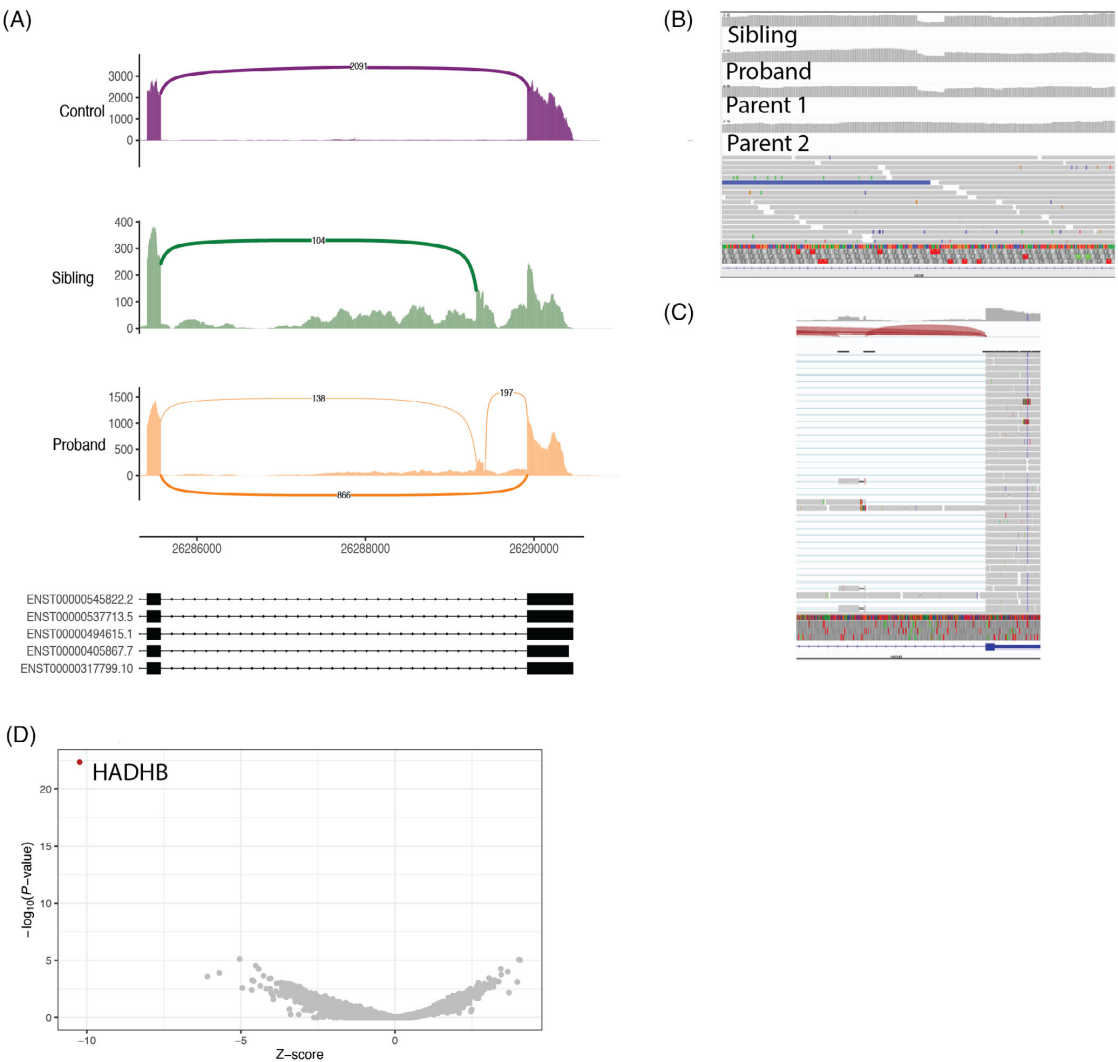
RNAseq identifies a second variant in *HADHB*. (A) Sashimi plot of RNAseq in cultured fibroblasts demonstrates a novel pseudoexon in the proband and sibling (the sibling RNAseq reads were not split properly, hence the pseudoexon acceptor usage shown without the donor). (B) presence of the 17 bp deletion in genome sequencing and segregation with affected individuals. (C) RNAseq reads of the pseudoexon in proband showing the 17 bp deletion. (D) OUTRIDER finds HADHB to be signficantly underexpressed compared with control samples.

RNAseq derived from fibroblasts was pursued for both proband and sibling. Inspection of *HADHB* identified an aberrant pseudoexon in the terminal intron (intron 15) of both the proband and sibling but not in control samples (Figure 2A). Further inspection of the novel pseudoexon reads revealed a 17 base pair deletion (Figure 2B,C). Subsequent analysis of DNA in the terminal intron of *HADHB* confirmed the 17 base pair deletion to be NM_000183.3:c.1390‐515_1390‐499del (rs1673175178). This deletion is not reported in ClinVar and has an allele frequency of 0.000006583 (1/151910 alleles) in gnomAD v4.0.0, with no homozygotes reported.^19^ It has a CADD score of 7.77 and a spliceAI score of 0.270 (donor gain).^22^ This is classified as a VUS, meeting PM2_supporting, PM3, and PP4.^20^, ^21^ We think the deletion acts to create a novel donor motif, resulting in aberrant splicing. The resultant pseudoexon contains 2 premature stop codons and 8 amino acids into the pseudoexon reading frame. In addition, *HADHB* is reported as an underexpressed outlier for the sibling, suggesting that this pseudoexon‐containing transcript is degraded (Figure 2D).

### 3.5 RT‐PCR confirmation

**FIGURE 3:**
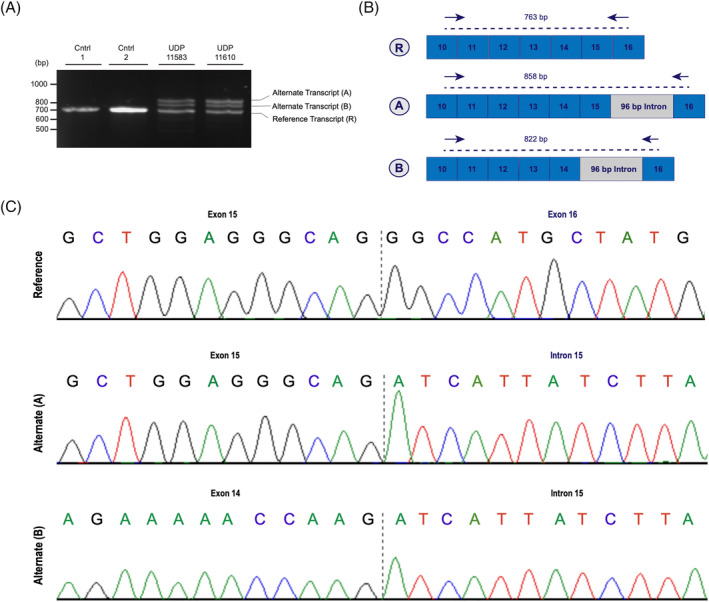
RT‐PCR confirms aberrant splicing events. (A) Agarose gel electrophoresis of cDNA samples from both patients and controls. Both affected individuals show two additional bands (A and B) that are higher molecular weight than the predicted reference transcript (R). (B) A schema that represents splicing isoforms. The reference transcript (R) spans exons 11–15, while alternative transcript (A) spans exon 11–15 and includes at 96 bp intronic insertion that excludes the c.1390‐515_1390‐499del, atcattatcttaacctgtgtagaatagtgaagaagggacaagcccagcctttggaatcatctagacctggtttcaaatcctggccctgtgatttgg. Alternative transcript (B) appears to skip exon 15 and include the same 96 bp intronic insertion. (C) Sanger sequencing (5′→3′) of cloned RT‐PCR products shows utilization of a new splice acceptor site following either exon 14 or 15.

cDNA from the proband and affected sibling compared to two unaffected controls to confirm the effect of the 17 bp deletion on splicing. RT‐PCR and subsequent gel electrophoresis revealed two bands in the patient and affected sibling that were higher molecular weight than the expected 763 bp band and not observed in the unaffected controls (Figure 3A). The RT‐PCR product was cloned and selected clones were screened on a gel to elicit frequency of the alternate transcripts compared to the expected (Figure S1). The reference transcript was observed in 12 (60%) of the proband clones and 12 (46%) of the affected sibling. A 858 bp transcript with the 96 bp intron 15 inclusion was observed in 8 (40%) of the proband's selected clones and 7 (27%) of the affected sibling's clones. On the gel, there is another band which could be an alternate transcript. Sequencing of this band revealed a 822 bp isoform that skips exon 15 but includes the 96 bp intron 15 inclusion (Figure 3B). Sanger sequencing shows utilization of a new splice acceptor site following either exon 14 or 15 (Figure 3C). The inclusion of the intron 15 is predicted to lead to a frameshift and the incorporation of eight amino acids followed by a premature stop codon. These results provide evidence of alternative splicing in both affected individuals leading to the generation of alternative transcripts in *HADHB*.

## 4 DISCUSSION

Arriving at a definitive diagnosis involves synthesizing clinical, biochemical, and molecular findings. Here we present two siblings with TFP and a single heterozygous variant in *HADHB*. Although RNAseq identified a convincing second variant in *HADHB* which was validated with RT‐PCR, several aspects of the clinical phenotype were unusual. Biallelic variants in HADHB adequately explained some features including hypoglycemia, retinopathy, hepatomegaly, and hypoparathyroidism. Additional clinical features including nephrotic syndrome, pancytopenia, and pulmonary involvement left us searching for secondary genetic explanations. However, genome sequencing did not identify additional candidate variants.

Literature review revealed a few recent cases reporting similar unusual features in TFP patients. Kumar et al. discussed a child with a homozygous variant in *HADHA* that presented with collapsing glomerulopathy, a variant of FSGS. Notably, hypoparathyroidism, anemia, and neutropenia were also seen in this case.^23^ This was followed by a case report and literature review in 2021 of another patient with LCHAD due to compound heterozygous variants in *HADHB*. This individual's course was also complicated by hypoparathyroidism, neutropenia, and nephrotic syndrome. Out of 157 cases, hypoparathyroidism was seen in 9, renal involvement in 6 with 1 case of nephrotic syndrome, and 3 cases of hematologic involvement.^24^

RNAseq played a crucial role in both identification of a second variant and functional validation of this deep intronic 17 base pair deletion. Without the integrative analysis combining RNAseq and genome sequencing, it would have been difficult to prioritize which deep intronic variants to assess further. This case adds to the growing number of pathologic deep intronic variants identified and highlights the role of non‐coding variants in inherited metabolic disease.^25^ Furthermore, as gene therapies continue to move closer to clinical practice, this case is the one that would potentially have been amenable to splice altering therapies. The rise in splice altering therapies as a viable treatment option makes the identification of non‐coding causative variants even more important.^26^

When patients do not fit the typical phenotype for a given diagnosis, there is always the question of phenotypic expansion or a second disease that is adding to the clinical features. While the literature supports the findings in these siblings are uncommon but associated with TFP, the use of genome sequencing in this case allows even greater confidence that these findings can be explained by a single diagnosis. One additional possibility that genome sequencing allows is for the identification of modifiers. For instance, in TFP, it is possible that these few patients with similar presentations share other more common variants that may not be independently pathogenic but contribute to their shared features. The increasing availability of the genome sequencing could allow for additional cohort studies in the future.

This case highlights the importance of iterative reanalysis of both the clinical and molecular findings in reaching a diagnosis. Both the UDN and referring care providers played an instrumental role. The UDN was able to utilize emerging technologies to find a previously undetected variant. The referring care providers were able to update the phenotype and unify the molecular findings with the clinical diagnosis through careful literature review. Ongoing communication led to a unified molecular, biochemical, and clinical diagnosis in this family.

## CONFLICT OF INTEREST STATEMENT

The authors declare no conflicts of interest.

## INFORMED CONSENT

All procedures followed were in accordance with the ethical standards of the responsible committee on human experimentation (institutional and national) and with the Helsinki Declaration of 1975, as revised in 2000 (5). Informed consent was obtained from all patients for being included in the study. Proof that informed consent was obtained is available upon request.

## ANIMAL RIGHTS

This article does not contain any studies with animal subjects performed by the any of the authors.

## Supporting information


**Supplemental Figure S1.** Results of Sanger sequencing of cloned RT‐PCR products.

**Supplemental Figure S2.** Quality control data (generated by RNA‐SeQC) for strand specificity. The sibling sample is highlighted. Other samples depicted are controls.

**Data S1.** Supporting Information.

## Data Availability

Genomic information for and RNAseq dataset for the subjects and family members were deposited in dbGap (phs001232.v5.p2).
