# Supplementary figures and images for: Mitochondrial trifunctional protein deficiency caused by a deep intronic deletion leading to aberrant splicing
Source: JIMD Rep. 2024 Dec 16;66(1):e12459. doi: 10.1002/jmd2.12459 (PMC11667764; doi:10.1002/jmd2.12459)

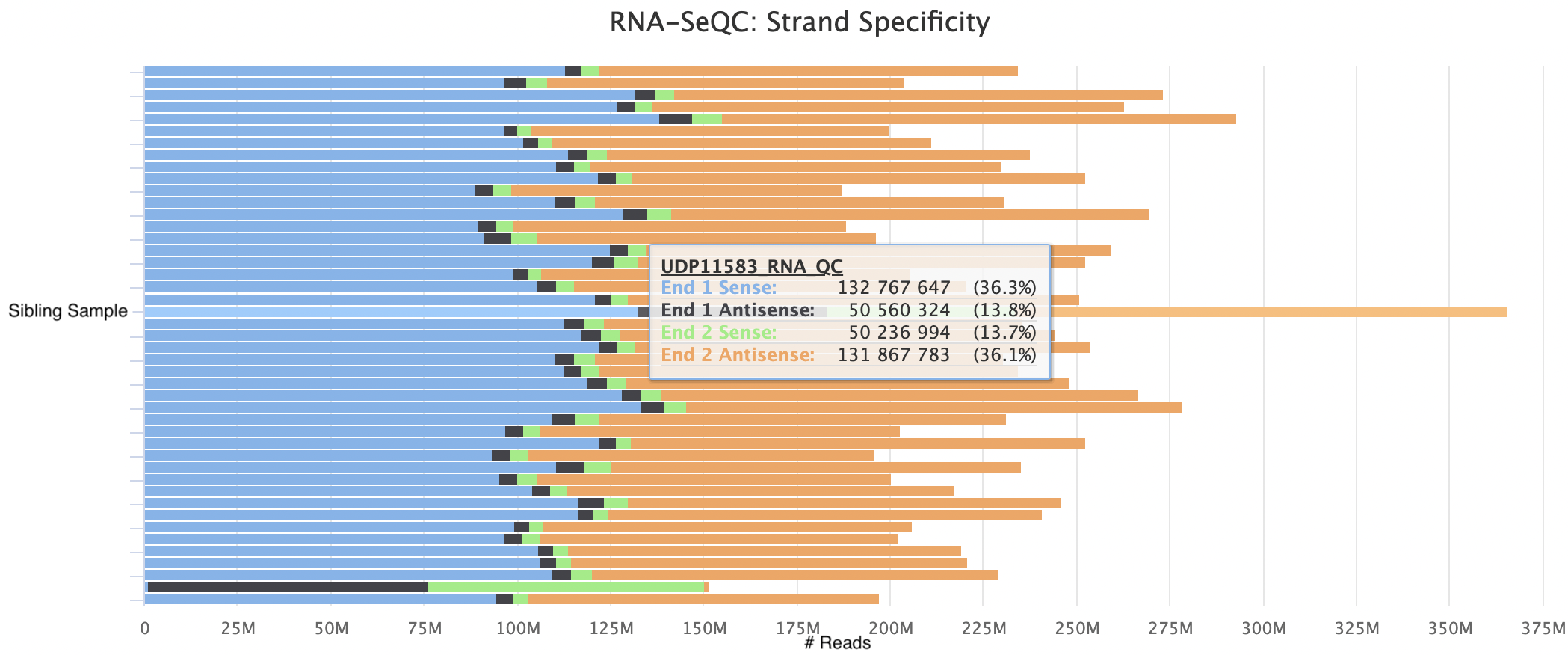

Supplement: Supplementary file 2 — Supplemental Figure S2. Quality control data (generated by RNA‐SeQC) for strand specificity. The sibling sample is highlighted. Other samples depicted are controls. [file JMD2-66-e12459-s002.tiff]
